# Nanoemulsion and nanogel containing *Artemisia dracunculus* essential oil; larvicidal effect and antibacterial activity

**DOI:** 10.1186/s13104-022-06135-8

**Published:** 2022-08-12

**Authors:** Mahmoud Osanloo, Samira Firooziyan, Abbas Abdollahi, Shekoufeh Hatami, Amene Nematollahi, Narges Elahi, Elham Zarenezhad

**Affiliations:** 1 Department of Medical Nanotechnology, School of Advanced Technologies in Medicine, Fasa University of Medical Sciences, Fasa, Iran; 2 Medical Entomology, Disease Control Unit, Urmia Health Center, Urmia University of Medical Sciences, Urmia, Iran; 3 Department of Microbiology, School of Medicine, Fasa University of Medical Sciences, Fasa, Iran; 4 Department of Biochemistry, School of Medicine, Fasa University of Medical Science, Fasa, Iran; 5 Department of Food Safety and Hygiene, School of Health, Fasa University of Medical Sciences, Fasa, Iran; 6 Department of Tissue Engineering, School of Advanced Technologies in Medicine, Fasa University of Medical Sciences, Fasa, Iran; 7 Noncommunicable Disease Research Center, Fasa University of Medical Sciences, Fasa, Iran

**Keywords:** Nanotechnology, Infection diseases, Vector-borne disease, Malaria, *Anopheles stephensi*

## Abstract

**Objective:**

Microbial infections and mosquito-borne diseases such as malaria, with 627 k deaths in 2020, are still major public health challenges.

**Results:**

This study prepared nanoemulsion and nanogel containing *Artemisia dracunculus* essential oil. ATR-FTIR analysis (Attenuated Total Reflection-Fourier Transform InfraRed) confirmed the successful loading of the essential oil in nanoemulsion and nanogel. LC50 values (Lethal Concentration 50%) of nanogel and nanoemulsion against *Anopheles stephensi* larvae were obtained as 6.68 (2–19 µg/mL) and 13.53 (7–25 µg/mL). Besides, the growth of *Staphylococcus aureus* after treatment with 5000 μg/mL nanogel and nanoemulsion was reduced by ~ 70%. However, about 20% growth of *Pseudomonas aeruginosa* was reduced at this dose. Considering the proper efficacy of the nanogel as a larvicide and proper antibacterial effect against *S. aureus*, it could be considered for further investigations against other mosquitoes’ larvae and gram-positive bacteria.

## Introduction

Malaria is preventable, but it is still the most dreadful vector-borne disease; according to the latest report of WHO, there were about 241 million cases and 627,000 deaths worldwide only in 2020 [1]. *Anopheles stephensi* Liston is one of the most important malaria vectors in the Middle East and South Asia [2, 3]. Besides, larviciding in 55 countries is one of the most important malaria control methods [1]. However, excessive chemical larvicides have threatened human and environmental health and caused resistance in vectors [4].

Moreover, microbial infections are another health challenge. *Staphylococcus aureus* (gram-positive) and *Pseudomonas aeruginosa* (gram-negative) are two common opportunistic bacteria that cause several infections like skin maladies such as pain, swelling, and skin color in humans [5, 6]. Microbial drug resistance and side effects of chemical drugs are other new emerging challenges of the health systems [7, 8]. Therefore, developing new drugs and insecticides with fewer side effects is critical.

For thousands of years, plant-derived extracts and essential oils (EOs) have been widely used as insecticides and natural antibiotics [9, 10]. Moreover, the efficacy of some of them is promising, e.g., *Artemisia dracunculus* EO with LC50 11.36 µg/mL against *A. stephensi* [11]. Therefore, this EO was classified in the active category that can be a good alternative to synthetic larvicides [12]. Furthermore*, A. dracunculus* EO also possesses anti-inflammatory, anticancer, antifungal, and antibacterial effects [13, 14].

Nowadays, it is generally accepted that formulating the EOs in nanoemulsion and nanogel dosage forms improves their stability and efficacy [15, 16]. Here, for the first time, the larvicidal effects of a nanogel (containing *A. dracunculus* EO) were investigated against *A. stephensi* and compared to its nanoemulsion. Moreover, their antibacterial effects were investigated against *S. aureus* and *P. aeruginosa*.

## Main text

### Methods and materials

#### Preparation and characterization of the nanoemulsion and nanoemulsion-based nanogel

Bark-extracted *A. dracunculus* EO was purchased from Zardband Pharmaceuticals Company (Iran). The nanoemulsion was prepared as follows; the EO (100 μL) and tween 20 (300 μL) was first mixed at 2000 rpm for 3 min at ambient temperature to form a homogeneous solution. Distilled water was then added to the mixture up to desired volume (5000 μL) and was stirred for another 40 min at 2000 rpm. Finally, the prepared nanoemulsion's droplet sizes and droplet size distribution were investigated utilizing a Dynamic Light Scattering (DLS) apparatus (K-One NANO- Ltd. Korea). Droplet size distribution was computed as d90-d10/d50, where d10, 50, and 90 are percentiles of droplets with diameters less than these values.

The nanoemulsion was gelified by adding 3.5% w/v carboxymethylcellulose; the mixture was stirred at a mild speed (180 rpm) for 4 h. Moreover, nanoemulsion (-oil) and nanogel (-oil) were prepared using the same process, only without the EO.

The viscosity of the prepared nanogel was assayed at different shear rates at 25° C under atmospheric pressure (Rheometer machine model MCR-302, Anton Paar, Austria). Besides, ATR-FTIR analysis was used to investigate the successful loading of the EO in the nanoemulsion and nanogel. Spectra of the EO, nanoemulsion (-oil), nanoemulsion, nanogel (-oil), and nanogels were recorded in a wavenumber range of 400–3900 cm^−1^ using a spectrometer (Tensor II model, Bruker Co, Germany).

#### Evaluation of larvicidal activity

In the current study, *A. stephensi* late-3rd or young-4th instar larvae were used; they were reared and maintained at 29 ± 2 °C with 70 ± 5% humidity at Urmia University of Medical Sciences (Iran). Mosquitoes are not exposed to any insecticides for more than 10 years. According to the WHO guideline, the larvicidal activity was done with a slight modification [17]. Briefly, beakers containing 200 mL of no-chlorine water and 25 larvae were first prepared. The larvicidal effects of nanoemulsion and nanogel were then investigated at 6.3, 12.5, 25, 50, and 100 µg/mL*.* Larval mortality after 24 h exposure was counted, while the larvae were not fed during the test. The larvae were exposed to 1.5 mL ethanol and nanoemulsion (-oil) and nanogel (-oil) in the control and negative control group.

#### Evaluation of antibacterial activity

The antibacterial activity of nanoemulsion and nanogel against *S aureus* (ATCC 25,923) and *P aeruginosa* (ATCC 27,853) were investigated using ATCC100 standard method [18]. Briefly, 4 mL of each bacterial suspension (2 × 10^5^ CFU/mL) was first poured into 5 cm plates separately. Antibacterial effects of nanoemulsion and nanogel were then investigated at 1250, 2500, and 5000 µg/mL. The treated plates were incubated at 37 °C for 24 h, and 10 μL-microbial suspensions were cultured on agar plates and incubated for 24 h. The number of grown colonies on the plates was counted and compared to the control sample. The control groups were not treated, and the negative control group was treated with nanoemulsion (-oil) and nanogel (-oil). Growth (%) of bacteria in each plate was calculated as (*CFU sample /CFU control*) × 100.

#### Statistical analyses

Three replicates were carried out for all tests, and final values were given as mean ± standard deviations. The samples were compared with SPSS software using one-way ANOVA with at least a confidence interval of 95%.

### Results

#### Prepared nanoemulsion and nanogel

**Fig. 1:**
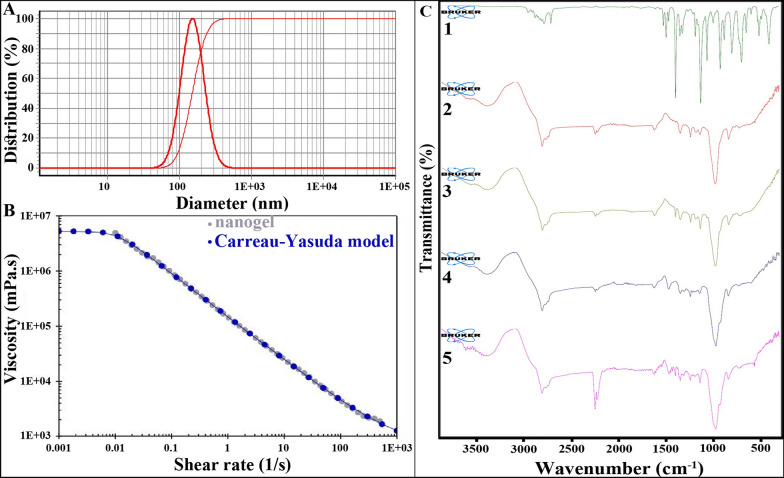
**A** DLS profile of the nanoemulsion containing *A. dracunculus *EO with a droplet size of 152 ± 6, **B** viscosity cure of the nanogel containing the EO is fully fitted with the Carreau-Yasuda model, **C** ATR-FTIR spectra of 1: *A. dracunculus *EO, 2: nanoemulsion (-oil), 3: nanoemulsion containing the EO, 4: nanogel (-oil), and 5: nanogel containing the EO

DLS profile of the nanoemulsion with a droplet size of 152 ± 6 nm is shown in Fig. 1A. The nanoemulsion had narrow particle size distribution as its droplet size distribution was 0.98; its single sharp peak also confirmed its uniform droplet size [19]. The viscosity of nanogel at different shear rates (1/s) is fully fitted with the Carreau-Yasuda models (Fig. 1B). This well-known empirical equation has been used for non-Newtonian fluids; viscosity is decreased with increasing shear rates [20].

Besides, successful loading of the EO in nanoemulsion and nanogel was confirmed using ATR-FTIR analysis (Fig. 1C). The spectrum of *A. dracunculus* EO showed the bands at 3061 and 3028 cm^−1^ related to = C-H. The bands at 3076, 3001, 2933, 2096, and 2834 cm^−1^ displayed –CH stretching vibration in SP^3^ and SP^2^. Besides, the bands at 1727 and 1638 cm^−1^ can be related to the stretching vibration carbonyl C = O group. The peak at 1243 cm^−1^ corresponds to the stretching vibrations of C-O. The peak at 1035 cm^−1^ is assigned to C-H bending absorption, and the peak at 808 cm^−1^ is attributed to benzene rings C-H vibration absorption.

The spectrum of nanoemulsion (-oil) showed broadband between 3300 to 3600 cm^−1^ can be attributed to the presence of hydroxyl group due to hydrogen bonding. Besides, the peak at 2923 cm^−1^ is attributed to C-H stretching in tween. Moreover, the peak at 1732 cm^−1^ corresponds to C = O stretching exhibiting ester groups in tween 20. The sharp band at 1088 cm^−1^ is assigned to C-O stretching vibration.

In the spectrum of nanoemulsion, the broadbands at about 3200 to 3600 cm^−1^ are related to the hydroxyl group due to hydrogen bonding. The absorbance band at 2923 cm^−1^ showed CH stretching vibration in tween 20 and EO. Besides, the band at 1734 cm^−1^ can be related to the carbonyl group in the EO and tween 20. The band at 1457 cm^−1^ is related to CH_2_ bending in the EO and tween 20. All the other characteristic bands appear in the spectra of the EO and nanoemulsion (-oil).

The spectrum of nanogel (-oil) showed the broadband at about 3200 to 3600 cm^−1^ is attributed to the OH group due to hydrogen bonding. The band at 1738 cm^−1^ showed C = O stretching that represents the carbonyl group in CMC and tween 20. The characteristic band at 1579 cm^−1^ is attributed to the carboxyl group in CMC.

In the spectrum of nanogel, the broadband at around 3200 to 3600 cm^−1^ is attributed to the OH group due to hydrogen bonding. The interaction between CMC and the EO during gel formation is related to the preparation of hydrogen bonding. The formation of hydrogen bonds increases the degree of polarization of chemical bonds. Besides, the peak at 1733 cm^−1^ exhibited carbonyl stretching that confirmed the carbonyl group in CMC, tween 20, and the EO. The peak at 1579 cm^−1^ corresponded to the carboxyl group in CMC. All the other characteristic peaks appear in the EO and nanogel (-oil) spectra at the same wavenumber.

#### Larvicidal effect of the nanoemulsion and nanogel

**Fig. 2:**
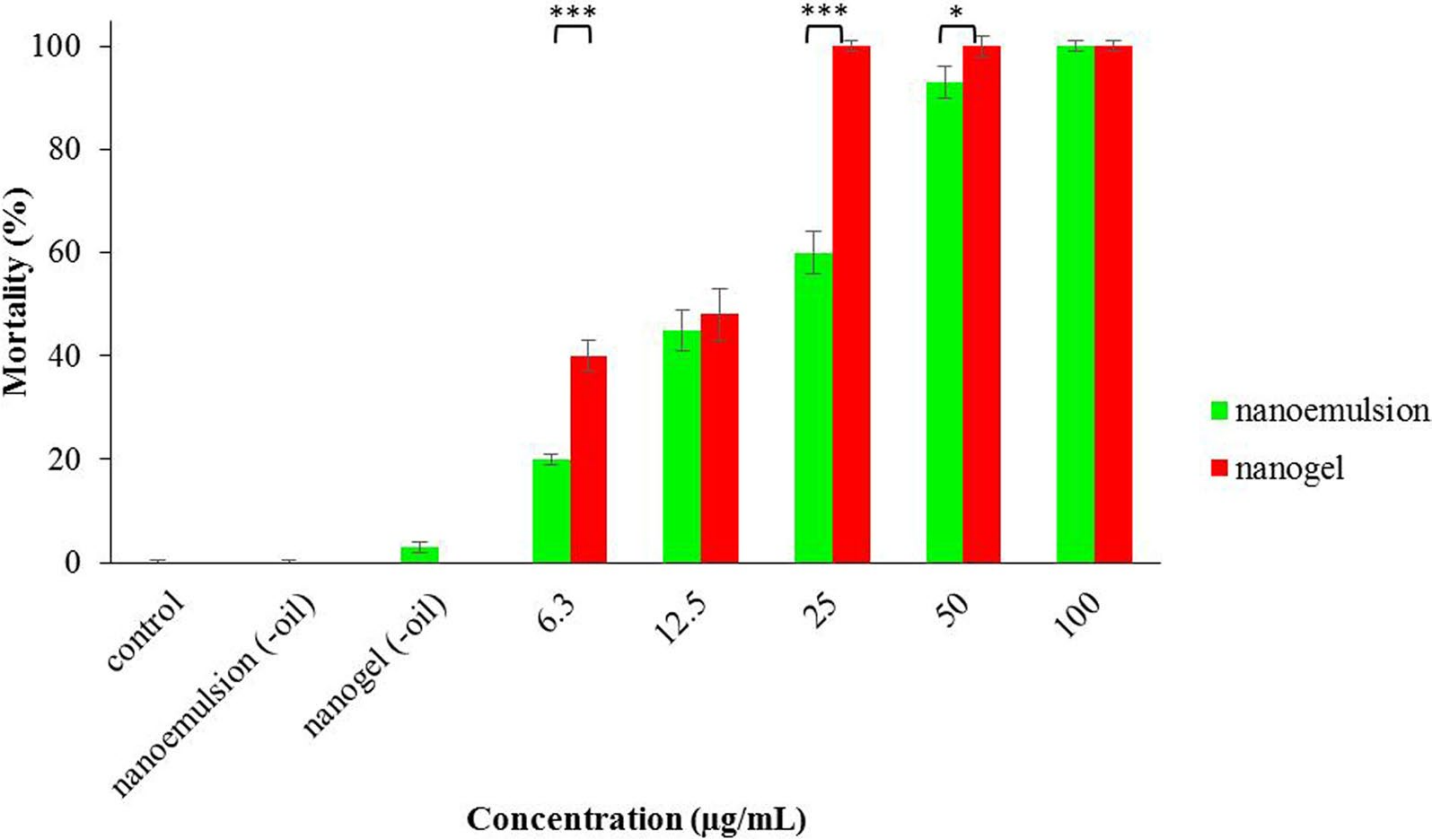
Larvicidal effects of nanoemulsion and nanogel containing *A. dracunculus *EO against A. stephensi. *P < 0.05 and ***: P < 0.001

Larvicidal effects of nanoemulsion and nanogel against *A. stephensi* are given in Fig. 2. Dose-dependent responses are observed in their efficacy; however, the nanogel with LC50 6.6 (2–19) µg/mL was more potent than the nanoemulsion with LC50 13.5 (7–25) µg/mL. Besides, nanogel was significantly more potent than nanoemulsion at 6.3 µg/mL (P < 0.001), 12.5 µg/mL (P < 0.001), and 50 µg/mL (P < 0.028). Interestingly, perfect efficacy (100% larval mortality) was observed at 25, 50, and 100 µg/mL nanogel. Moreover, nanoemulsion (-oil) and nanogel (-oil) with 0 and 3% larval mortality did not affect larvae.

#### Antibacterial effects of the nanoemulsion and nanogel

**Fig. 3:**
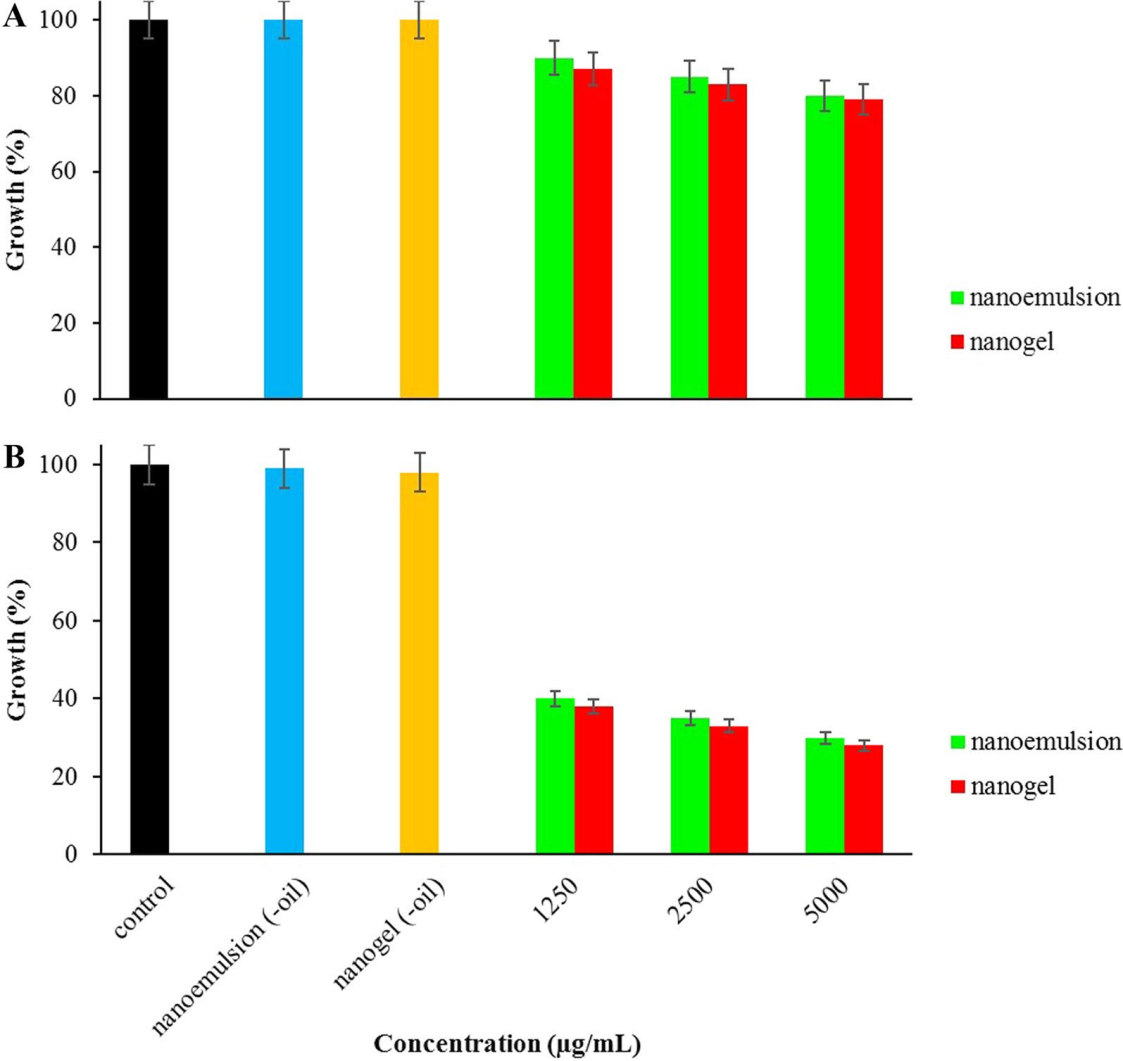
Antibacterial effects of nanoemulsion and nanogel containing *A. dracunculus *EO against **A** *P. aeruginosa *and **B** *S. aureu*s

The antibacterial effect of nanoemulsion and nanogel against *P. aeruginosa* and *S. aureu*s are shown in Fig. 3(A and B). The efficacy of nanogel was more potent than nanoemulsion; however, this difference was not significant at all examined concentrations (P > 0.05). The best efficacy (~ 20% growth inhibitory) against *P. aeruginosa* was observed at 5000 μg/mL nanogel and nanoemulsion. However, 70% growth inhibitory was observed at this point against *S. aureu*s. Moreover, nanoemulsion (-oil) and nanogel (-oil) did not affect bacterial growth.

## Discussions

The preparation of nanostructures-loaded EOs has received more attention as a promising approach to developing new natural insecticides and drugs [15]. Efficacy of such mentioned nanosystems against important mosquitoes’ larvae, including *Aedes* (spp.), *Anopheles* (spp.), and *Culex* (spp.), have been reported in the literature. For instance, LC50 of *Lippia alba* EO nanoemulsion against *A. aegypti* was 31.02 μg/mL [21]. LC50 value of nanoemulsion of *Mentha piperita* EO against *C. Pipiens* was 31.24 μg/mL [22]. Besides, nanocrystal emulsion of *Ficus glomerata* EO with LC50 17 μg/mL against *A. stephensi* showed proper efficacy [23]. The current study investigated the larvicidal effect of nanogel containing *A. dracunculus* EO for the first time against *A. stephensi*. Its efficacy was more potent than nanoemulsion due to its proper stability and sustained release profile. Nanogels with soft tissue, high drug loading capacity, biocompatibility, biodegradability, good swelling ability, and structural stability have recently received more attention [24–26].Article structure: Kindly check whether the section headings have been identified correctly and amend if any.It is ok, thanks.

Bacterial infections may cause serious diseases in humans and animals [27, 28]. In the current research, the efficacy of both nanoemulsion and nanogel against *S. aureus* (gram-positive) was more than *P. aeruginosa* (gram-negative). This agrees with the literature; gram-negative bacteria with an extra outer membrane are more resistant to antibiotics than Gram-positive bacteria [29]. However, the Gram-positive bacteria cell wall structure allows hydrophobic molecules to penetrate the cells easily [30].

Some reports on the antibacterial effects of nanoemulsion and nanogel containing EOs have been found in the literature. For instance, thyme EO nanoemulsion reduced *E. coli* populations by 3.28–4.13 log CFU/mL [31]. Moreover, the growth of *P. aeruginosa* after treatment with 2500 and 5000 µg/mL of nanogel containing *Mentha longifolia* EO was reduced by 5 and 90%. On the other hand, the growth of *S. aureus* after treatment with such doses was reduced by 3 and 100% [6].

## Conclusions

A comprehensive comparison was carried out on the efficacy of nanoemulsion and nanogel containing *A. dracunculus,* EO. The nanogel at 25, 50, and 100 µg/mL concentrations showed perfect larvicidal effects on *A. stephensi*. Moreover, the antibacterial properties of the nanoemulsion and nanogel were equal to each other and showed better efficacy against *S. aureus* than *P. aeruginosa*.

## Limitations

The efficacy of the nanoemulsion and nanogel could be investigated against other important mosquitoes’ larvae. In addition, it is recommended to investigate the efficacy of the nanoemulsion and nanogel on clinical isolated bacteria strains.

## Data Availability

All data generated or analyzed during this study are available from the corresponding author on reasonable request.

## Abbreviations

EO: Essential Oil
ATR-FTIR: Attenuated Total Reflection-Fourier Transform InfraRed
DLS: Dynamic Light Scattering
LC50: Lethal Concentration 50
